# Genomic similarity to quantitatively evaluate the reassortment potential of H7N9 with other subtypes of avian influenza viruses

**DOI:** 10.3389/fcimb.2026.1777911

**Published:** 2026-03-27

**Authors:** Yuan Gao, Ying Liu, Zi-Wei Cao, Zhi-Hong Ma, Ying Wang, Yuan Cao, Tao Jiang

**Affiliations:** 1 Department of Basic Medical Sciences, The 960th Hospital of People’s Liberation Army (PLA), Jinan, China; 2 School of Basic Medical Science, Inner Mongolia Medical University, Hohhot, China; 3 State Key Laboratory of Pathogen and Biosecurity, Academy of Military Medical Sciences, Beijing, China; 4 Department of Pulmonary and Critical Care Medicine, The 960th Hospital of PLA, Jinan, China

**Keywords:** genomic similarity, H7N9, host distribution, predictive framework, reassortment potential, spatiotemporal distribution

## Abstract

**Introduction:**

The H7N9 influenza virus poses a significant public health threat due to its potential for reassortment and cross-species transmission. This study aims to systematically evaluate the genomic similarity between H7N9 and other influenza A subtypes to identify strains with high reassortment potential and characterize their spatiotemporal and host distribution patterns.

**Methods:**

We analyzed nearly 4,000 H7N9 sequences from GISAID and NCBI, alongside representative sequences of other influenza A subtypes. Open reading frames were extracted, and a genomic similarity index was constructed using Euclidean distance, dot product, and cosine similarity measures, with weights optimized via principal component analysis. The index was applied to quantify inter-subtype similarity and predict reassortment-prone strains.

**Results:**

High sequence similarity was observed between H7N9 and cognate subtypes (e.g., H7N3, H15N9), with H7N3 exhibiting the highest similarity index (1.00). Validation using known reassortant strains, such as A/Yixing/805/2022 (H3N2), confirmed that strains with high reassortment potential showed significantly elevated similarity scores across all gene segments (p< 0.001). High-similarity outliers analysis identified 581 spillover events, temporally concentrated during 2014–2017, and spatially clustered in regions like the United States, Europe, and Hong Kong. Host analysis highlighted birds—especially chickens, ducks, and turkeys—as key reservoirs for reassortment.

**Discussion:**

The genomic similarity index effectively identifies influenza A subtypes with high reassortment potential, supported by retrospective validation and spatiotemporal congruence with documented outbreaks. The concentration of high-similarity strains in specific hosts and regions underscores the role of ecological factors in viral evolution. These findings provide a predictive framework for monitoring emergent reassortants and inform targeted surveillance strategies.

## 1 Introduction

Influenza A virus (IAV) has been proven to cause pandemics in human populations (Joseph et al., 2017). Historically, due to the adaptive evolution to human and the reassortment of IAVs from multiple hosts (Kalyar et al., 2024; Linster et al., 2019; Smith et al., 2009; Taubenberger et al., 2019; Taubenberger et al., 2005), most IAV subtypes originating from avian reservoirs (AIV) pose a strong potential pandemic risk to humans (Rimondi et al., 2018). AIVs are globally distributed in wild waterfowl, and they constantly evolve into new reassortant strains through live poultry trade, migration, and genetic recombination (Chang et al., 2025; Liu et al., 2014a). The H5, H7, and H9 subtypes have repeatedly crossed the species barrier to infect humans and mammals (Chang et al., 2025; Deng et al., 2017; Sun et al., 2014a). During the co-circulation of diverse subtypes within overlapping ecological niches, the segmented nature of the IAV RNA genome facilitates high-frequency gene segment exchange, yielding reassortant viruses with novel HA/NA combinations (Sun et al., 2014b; Yao et al., 2024). Given the continuous reassortment, cross-species transmission, and virulence evolution of avian influenza viruses, identifying early warning targets and pre-empting spillover events before the next IAV pandemic remains a critical global health priority (de Bruin et al., 2022).

The emergence of new HA/NA combinations and adaptive internal gene reassortment are prerequisites for crossing the species barrier and causing a global outbreak (Zhang et al., 2018). Consequently, owing to its high pathogenicity, enhanced affinity for human receptors, and sustained reassortment capability, the H7N9 subtype has emerged as one of the most formidable potential pandemic candidate viruses to date (He et al., 2023). The H7N9 influenza virus first broke out in the Yangtze River Delta region of China in early 2013 (Lee et al., 2013). It is a novel triple reassortant virus composed of genes from three avian viruses: H7 (likely from the Eurasian H7N3 avian influenza virus), N9 (likely from the H11N9 virus), and H9N2 (providing six internal genes) (Nicoll and Danielsson, 2013). This virus caused at least five waves of human outbreaks between 2013 and 2017. The five waves of H7N9 infection have resulted in more than 1,600 laboratory-confirmed human cases, with a mortality rate of about 40% (Quan et al., 2018; Tang and Wang, 2017). In early 2017, the virus further reassorted into a highly pathogenic strain (HP-H7N9), with the insertion of multiple basic amino acids at the hemagglutinin (HA) cleavage site (Daidoji et al., 2024; Zhang et al., 2018). About 10 amino acids from other H7 subtype viruses (such as H7N3) were inserted, increasing the mortality rate to over 50% and causing limited human-to-human transmission, posing a serious threat to public health (Deng et al., 2017; He et al., 2022, He et al., 2018; Zhao et al., 2023). In August 2022, a novel H3N2 virus was discovered in Yixing, China. This was a “major reassortment” across hosts and subtypes. The six internal gene segments (PB2, PB1, PA, NP, M, NS) of this virus were all derived from the human H7N9 virus (Clade 2b) detected in pigs, while only the HA and neuraminidase (NA) surface protein genes of the swine H3N2 virus were retained, causing a fatal influenza-associated encephalitis/encephalopathy (AIE) (Wang et al., 2025). Recent monitoring shows that the highly pathogenic segments of H7N9, such as PB2 and PA, are reassorting with low-pathogenic avian H10 and H11 subtypes (Bisset and Hoyne, 2020). Previously rare combinations such as H11N9, H7N6 and H7N2 have been detected in live poultry markets in East and South China (Bhat et al., 2022; Huang et al., 2024; Liu et al., 2022; Sun et al., 2021a; Wang et al., 2023). Faced with the established fact that the highly pathogenic gene module of H7N9 continues to export the “human adaptation-high lethality” core ability to foreign subtypes such as H10 and H11 and generates immune escape reassortants (Sun et al., 2021a), timely evaluating its potential epidemic risk and intercepting its cross-subtype reassortment pathways prior to the next human outbreak has become an urgent core priority for monitoring and prevention.

Traditional epidemiological surveillance methods mainly rely on case reports, laboratory tests, and expert experience (Li et al., 2021; Liu et al., 2014b; Su et al., 2021). Although these conventional approaches were instrumental during the early stages of outbreaks, they exhibit limited predictive accuracy regarding the complex evolutionary trajectories of emerging viruses. This is especially true for IAV, which achieves genetic diversity through high-frequency gene reassortment and low but existing homologous recombination, producing a variety of mutant subtypes that pose great harm to humans (Li et al., 2021). In recent years, artificial intelligence (AI) technology has shown great potential in the field of infectious disease modeling and risk prediction (Chin et al., 2014). By integrating data such as viral gene sequences, AI models can capture the complex nonlinear relationships of viral transmission, enabling dynamic simulation and early warning of future epidemic trends (Jiang et al., 2025; Li et al., 2022; Zhang et al., 2024). For example, the VaxSeer system, built based on protein language models and evolutionary algorithms, has been successfully applied to predict the strain dominance of IAV (such as H3N2 and H1N1), with significantly higher prediction accuracy than traditional methods (Shi et al., 2025). Our team has recently made significant progress both by employing traditional machine-learning methods to analyze viral nucleotide composition (Zeng et al., 2025) and by introducing the deep-learning framework HAIRANGE to predict the adaptive risk of H5N1 (Wei et al., 2025). These studies simulate reassortment events through computational models and have partially validated polymerase activity. Therefore, for H7N9, which lacks pre-existing immune barriers in humans, has frequent viral gene reassortment and mutation, and has high uncertainty in transmission ability and pathogenic characteristics (Zhang et al., 2025), it is urgent to construct a quantitative assessment model for the potential epidemic risk of H7N9. Due to the frequent reassortment of the eight RNA segments in the H7N9 genome and the high incidence of segment deletion/insertion, the length and sequence differences of the same gene in different strains are extremely large (Zhang et al., 2020). This inherent ‘sequence misalignment’ introduces a substantial number of gaps and positional shifts at the input level for traditional AI models. This not only amplifies the estimation error of evolutionary distance but also makes it difficult for deep learning that relies on position encoding to capture the real mutation-phenotype-transmissibility mapping relationship, thereby significantly increasing the difficulty of AI prediction of the potential epidemic risk of H7N9. However, the algorithmic quantitative assessment method used in this study does not require sequence alignment. This study, targeting the problem of quantitative assessment of potential epidemic risk, parses all viral genes into vectors and performs directional calculations on the distance relationships of sequences, thereby quantitatively assessing the epidemic risk of viruses. Based on this, it presents a panoramic view of the epidemic risk of H7N9, validates the reported strains with high reassortment potential such as A/Shanghai/02/2013 (H7N9) and A/Yixing/805/2022 (H3N2), and predicts potential strains with a high probability of reassortment in the future. This study provides an innovative method for the potential epidemic risk of H7N9 virus.

## 2 Materials and methods

### 2.1 Preparation and preprocessing of sequence data

As of October 31, 2025, 3,218 H7N9 strains covering all 8 gene segments of influenza A virus (IAV) were downloaded from the Global Initiative on Sharing All Influenza Data (GISAID) database, and 745 H7N9 strains were obtained from the National Center for Biotechnology Information (NCBI) database. The screening process was not restricted by host, collection time, or segment integrity. The 8 gene segments included polymerase basic protein 2 (PB2), polymerase basic protein 1 (PB1), polymerase acidic protein (PA), hemagglutinin (HA), nucleoprotein (NP), neuraminidase (NA), matrix protein 1 (M1), and non-structural protein 1 (NS1).

Full-length sequence samples were used for strain genome assembly and nucleotide composition analysis. The full sequences of all 8 IAV segments were spliced in the order of PB2→PB1→PA→HA→NP→NA→M1→NS1 to construct a complete viral genome coding sequence (CDS). Raw data were standardized as follows: deduplication was performed via sequence alignment; low-quality sequences (excessively long: >1.5 times the mean length, excessively short:<0.5 times the mean length, and sequences with N content ≥3%) were excluded during quality control; open reading frames (ORFs) were extracted using the Python-wrapped Prodigal tool (v2.6.3) (Hadfield et al., 2018; Hyatt et al., 2010). Only strains with complete 8 gene segments were retained, and finally 1,922 high-quality H7N9 strains were obtained for subsequent analysis.

A total of 100 reference IAV subtypes were downloaded from the NCBI Virus database. Specifically, for each subtype, we utilized the “NCBI Virus Assembly” module, which curates strains with fully assembled genomes. We sorted the records by collection date and strictly selected the earliest available strain possessing all 8 complete gene segments. The fundamental rationale for selecting the earliest strains was to establish an ancestral genomic baseline for each subtype, free from the confounding effects of recent, highly complex multi-host reassortment events. This source-tracing approach allows us to evaluate the deep evolutionary roots and intrinsic genetic compatibility between modern H7N9 and the foundational lineages of other subtypes. Detailed metadata for these 100 reference strains, including their subtype, host compositions, and collection dates, are provided in **Supplementary Table 1**. ORF extraction was performed using the same method as described above.

### 2.2 Encoding of IAV genes and calculation of inter-gene distance

The overall objective of this step is to transform variable-length viral gene sequences into fixed-length numerical vectors, enabling the quantitative assessment of genetic compatibility (reassortment potential) between H7N9 and reference subtypes without sequence alignment. The input data consisted of the full-length coding sequences (CDS) of individual gene segments. For these input sequences, a global k-mer frequency vectorization approach was utilized rather than a sliding window method. The overall occurrence frequencies of 6 different types of tetranucleotide pairs were quantitatively analyzed across the full-length segments, and the distribution characteristics of valid codon pairs (excluding stop codon pairs) were counted simultaneously to construct global feature vectors (Greenbaum et al., 2008). The final output CSV format files included sequence IDs and their corresponding high-dimensional feature vectors.

It is crucial to emphasize that all subsequent similarity calculations were strictly performed between homologous gene segments (e.g., comparing the PB2 segment vector of an H7N9 strain exclusively with the PB2 segment vector of a reference strain). Dot Product, Cosine Similarity, and Euclidean Distance were calculated for the encoded feature data of each segment from 1,922 H7N9 strains and 100 reference subtype IAVs, with the specific formulas as follows:

Dot Product (Wang et al., 2024):


$$A\cdot B=\sum_{i=1}^{n}a_{i}b_{i}$$

Cosine Similarity (Horwege et al., 2014):


$$\cos\left(\text{θ}\right)=\frac{A\cdot B}{\left|A\right|\left|B\right|}=\frac{\sum_{i=1}^{n}a_{i}b_{i}}{\sqrt{\sum_{i=1}^{n}a_{i}^{2}}\cdot \sqrt{\sum_{i=1}^{n}b_{i}^{2}}}$$

Euclidean Distance (Sun et al., 2021b):


$$d\left(A,B\right)=\sqrt{\sum_{i=1}^{n}{\left(a_{i}-b_{i}\right)}^{2}}$$

Where n represents the total number of dimensions in the feature vector, and $a_{i}$ and $b_{i}$ represent the occurrence frequency of the i-th feature (e.g., a specific dinucleotide or codon pair) in the genomic sequence vector A (the target H7N9 strain) and vector B (the reference subtype strain), respectively. In this context, a higher similarity score calculated from these vectors represents a closer k-mer composition pattern. Biologically, this indicates higher genetic compatibility and shared evolutionary signatures between the evaluated segments, which strongly correlates with potential reassortment viability.

### 2.3 Calculation of similarity index for IAV genes

The calculated Dot Product was subjected to Min-Max normalization (value range mapped to 0–1):


$$dot_{norm}=\frac{dot-dot_{min}}{dot_{max}-dot_{min}}$$

Cosine Similarity was directly used for subsequent analysis as it is positively correlated with sequence similarity and has a value range of 0–1;

the Euclidean Distance was inversely transformed to align with the similarity trend, followed by normalization:


$$euc_{sim}=\frac{euc_{max}-euc}{euc_{max}-euc_{min}}$$

Principal Component Analysis (PCA) (Coombes et al., 2020) was applied independently to each gene segment to extract the consensus evolutionary signal and avoid subjective weighting biases. For each segment, the first principal component (PC1) consistently captured the vast majority of the total variance (exceeding 90% across all eight segments) among the three metrics. This confirms the high inherent correlation of the chosen distance metrics and justifies using PC1 loadings to derive a robust consensus index. Therefore, the PC1 loadings ( $l_{i}$) of each respective segment were used to assign specific weights to the indicators (Dot Product, Cosine Similarity, and Euclidean Distance), allowing for a dynamic and data-driven similarity evaluation:


$$w_{i}=\frac{\left|l_{i}\right|}{\sum_{j=1}^{3}\left|l_{j}\right|}\;\left(i=cos,dot,euc\right)$$

Based on the optimal weight matrix, a Similarity Index was constructed between each H7N9 strain and each reference subtype strain:


$$SI=w_{cos}\cdot cos_{norm}+w_{dot}\cdot dot_{norm}+w_{euc}\cdot euc_{sim}$$

Each gene segment ultimately formed a data matrix of 1922×96 to 1922×100 (among the 100 reference subtypes, some strains only yielded 7 ORFs via the Prodigal tool, resulting in slight differences in matrix dimensions). A total of approximately 1.5376 million Similarity Indices were obtained across all segments, providing core data support for subsequent statistical analysis and graphing.

## 3 Results

### 3.1 H7N9 virus surveillance and risk distribution analysis

**Figure 1:**
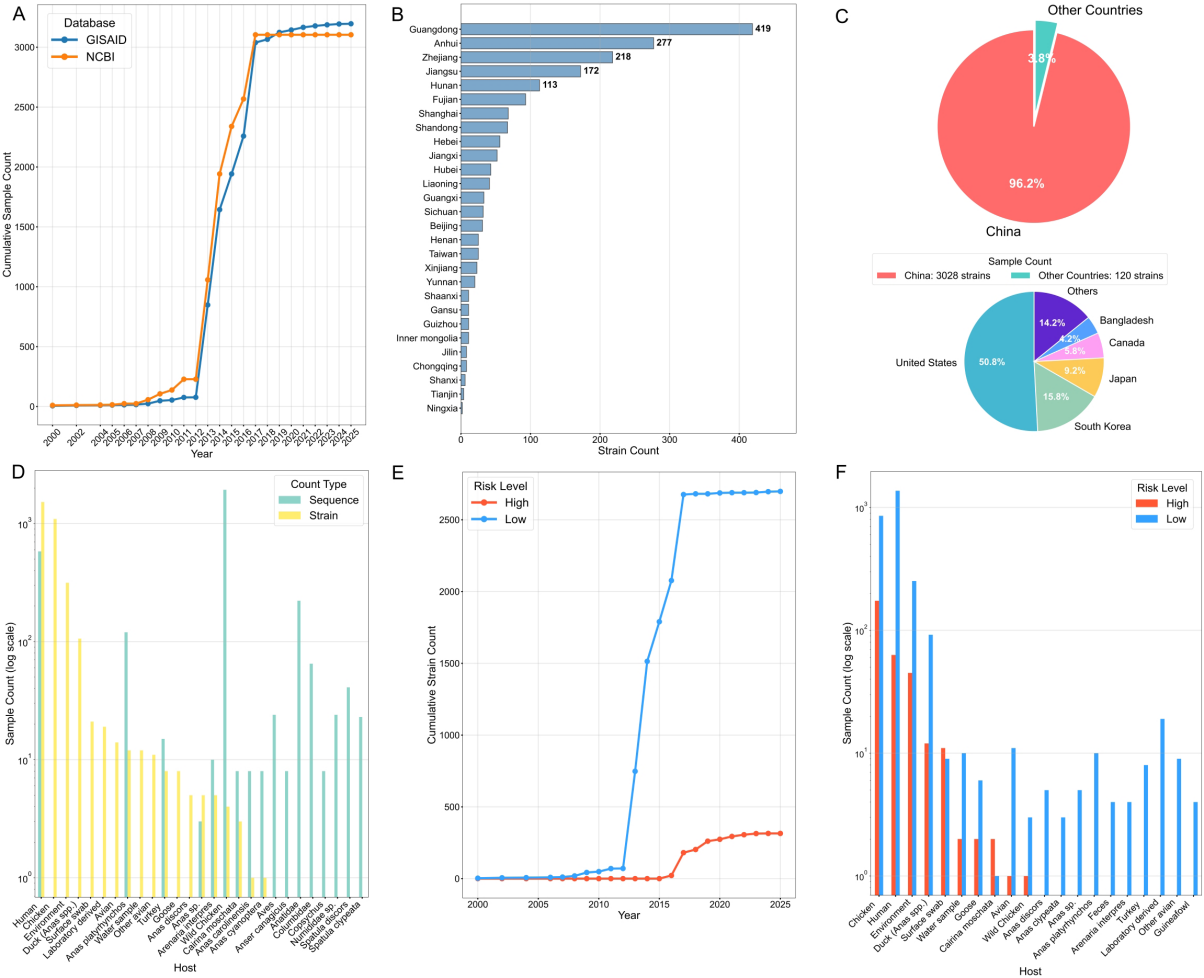
Temporal and spatial distribution, host lineage, and risk evolution of H7N9 virus. **(A)** Temporal trend of cumulative number of H7N9 virus sequence samples. The accumulation of sequences from the GISAID (blue) and NCBI (orange) databases has surged significantly since 2012-2013, with over 3,000 samples accumulated by 2020, indicating the continuous circulation of the virus and enhanced monitoring efforts. **(B)** Distribution of H7N9 virus strains in Chinese provinces. There is a distinct north-south difference, with Guangdong Province having the highest number of strains (419), followed by Anhui (277), Zhejiang (218), and Jiangsu (172), which are significantly higher than those in northern regions. **(C)** Global distribution of H7N9 virus. China accounts for an absolute majority (96.2%); among other regions globally, the United States (50.8%) reports the most cases, followed by Bangladesh, Canada, and other countries, with sporadic occurrences. **(D)** Number of H7N9 virus detections in different host samples (logarithmic scale). Human and environmental samples have the highest detection frequencies, and a large number of virus sequences are also detected in specific avian hosts (such as Chicken and Mallard). **(E)** Cumulative number of high- and low-potential H7N9 virus strains over time. The number of low-potential virus strains (blue) increased rapidly, peaking around 2015 and then stabilizing; high-potential strains (red) began to accumulate after 2015, with relatively steady but continuous growth. **(F)** Distribution of high- and low-potential H7N9 virus strains in different hosts (logarithmic scale). Chicken, Human, and Duck are key hosts carrying high- and low-potential H7N9 viruses, with sample counts much higher than those of other species, suggesting that they may play a central role in virus transmission and evolution.

This study provides a comprehensive analysis of the H7N9 virus, focusing on its temporal trends, geographical spread, and distribution across host species and risk categories. Temporal analysis of sequences from GISAID and NCBI revealed a pivotal increase in detection rates commencing in 2012-2013, with the cumulative number of sequences surpassing 3,000 and recent data from GISAID indicating ongoing circulation (**Figure 1A**). Within China, geographical analysis identified pronounced disparities, with southern and eastern provinces—notably Guangdong (419 strains), Anhui (277), Zhejiang (218), and Jiangsu (172)—being disproportionately affected compared to northern regions (**Figure 1B**). Globally, the geographical distribution of the virus was highly disproportionate, with China accounting for 96.2% of all reported strains (3,028/3,148); detections in other countries, such as the United States (~61 strains) and Japan (~19 strains), were sporadic (**Figure 1C**). Regarding host tropism, high detection frequencies were observed in human and environmental samples, with significant prevalence also noted in specific avian hosts like mallards and domestic chickens (**Figure 1D**). Temporal tracking of risk categories showed that low-potential strains proliferated rapidly, peaking around 2015 and subsequently plateauing, whereas high-potential strains have accumulated steadily since 2015 (**Figure 1E**). Most notably, risk-stratified host distribution pinpointed chicken, human and duck as critical reservoirs, as they alone harbored substantial quantities of both high- and low-potential variants, highlighting their potential centrality in the virus’s transmission ecology (**Figure 1F**).

### 3.2 A genomic similarity index for assessing H7N9 reassortment potential

**Figure 2:**
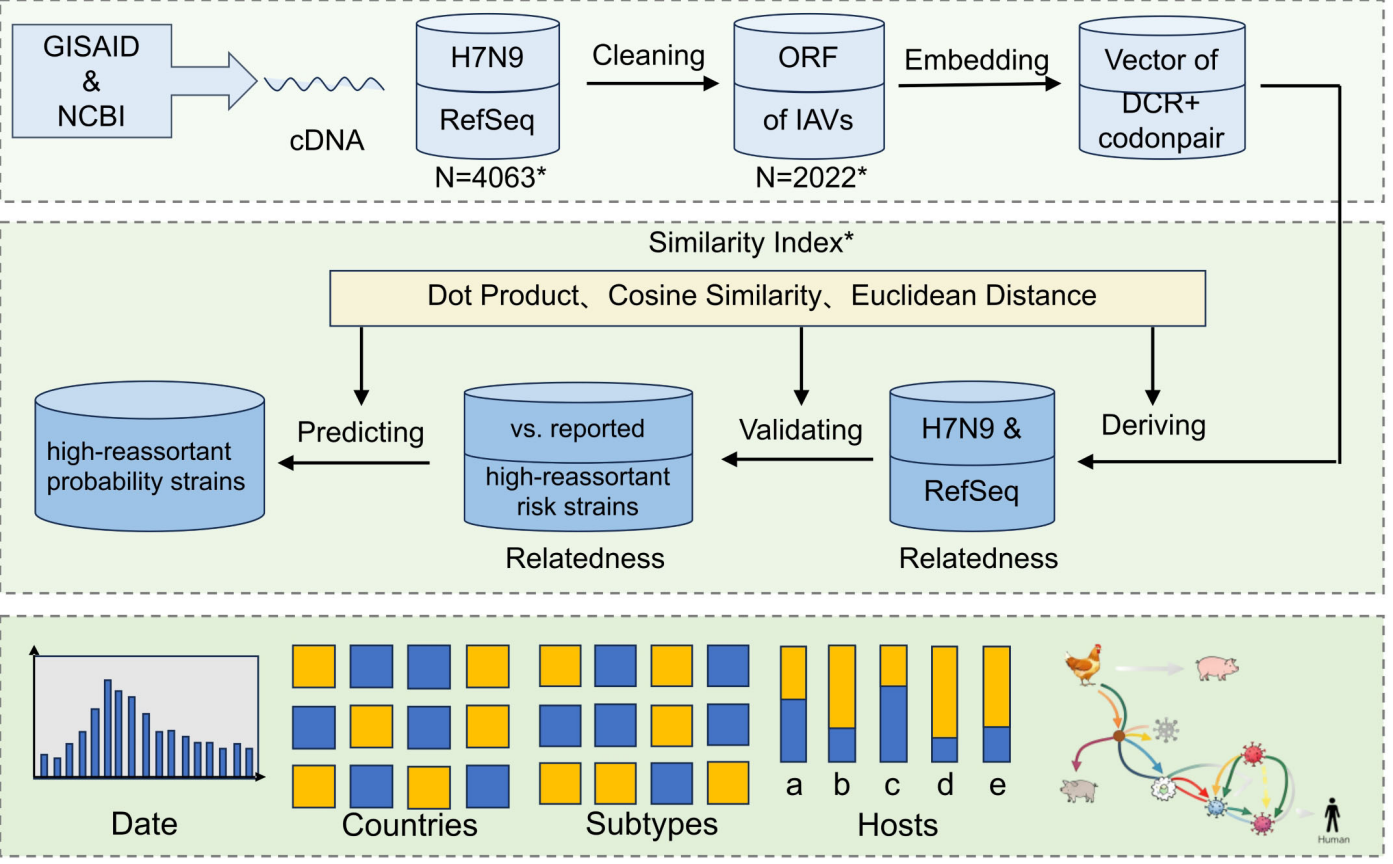
Bioinformatics workflow for reassortment potential assessment and prediction of H7N9 avian influenza virus. This study established a comprehensive computational biology pipeline to systematically assessing the reassortment potential of H7N9 virus. The workflow began with retrieving influenza A virus cDNA sequences from GISAID and NCBI databases, followed by rigorous data cleaning to generate H7N9 and representative strains of various subtypes (N = 2022). Open reading frames were extracted from all sequences, and an alignment-free global k-mer frequency vectorization approach was employed to calculate the overall occurrence frequencies of dinucleotide composition rules and codon pair bias features, generating global feature vectors. A genomic similarity index was computed using dot product, cosine similarity, and Euclidean distance algorithms, with reliability validated through comparison with reported high-reassortment potential strains. This index was subsequently applied to predict strains with high reassortment probability, followed by multidimensional distribution analysis. The results revealed distinct patterns across five key dimensions: temporal distribution (histogram showing concentration in specific years), geographical distribution (heatmap indicating regional clustering), subtype distribution (association matrix highlighting reassortment preferences), host distribution (bar chart statistics across multiple host types), and virus-host transmission networks (schematic diagram of cross-species pathways). These patterns collectively demonstrate that viral reassortment is influenced by spatiotemporal and host factors, providing critical insights for early warning of cross-species transmission. By integrating genomic features and multimodal similarity algorithms, this framework establishes a quantitative method for reassortment potential assessment, offering a novel paradigm for predicting influenza virus evolution and risk management. N = 4063*:H7N9 influenza A virus: GISAID = 3218 strains, NCBI = 745 strains; representative strains of various subtypes:100 strains. N = 2022*:H7N9 influenza A virus: 1922 strains; representative strains of various subtypes:100 strains. Similarity Index*:By calculating sequence similarity using dot product, cosine similarity, and Euclidean distance, and optimizing metric weights through principal component analysis, a genomic similarity index is constructed.

This study aimed to systematically assess the potential epidemic risk of H7N9 virus and predict influenza A virus subtypes with high reassortment potential, as well as their spatiotemporal and host distribution patterns. As shown in **Figure 2**, two datasets were retrieved from the GISAID and NCBI databases, comprising all available H7N9 sequences and representative strains of various subtypes. Following data cleaning, open reading frame extraction, and sequence coding, a global k-mer frequency vectorization scanning approach was employed to extract six categories of dinucleotide pair and codon pair features. Sequence similarities were computed using dot product, cosine similarity, and Euclidean distance measures (Grishin and Grishin, 2002; Yang and Wang, 2013). Principal component analysis was then utilized to optimize the weights of these indicators, leading to the construction of a genomic similarity index. Using this index, the study quantified the genomic similarity between H7N9 and reference strains of other subtypes, thereby identifying subtypes with a high propensity for reassortment with H7N9. To validate the reliability of the method, a retrospective analysis was performed on previously reported high-potential reassortant strains. The similarity index algorithm was subsequently applied to predict strains with a high probability of future reassortment. Statistical analysis of these predicted strains across temporal, spatial, and host dimensions demonstrated significant clustering in specific years, geographic regions, and host types, corroborating the hypothesis that viral reassortment is profoundly shaped by intersecting ecological and epidemiological factors.

### 3.3 Analysis of sequence similarity and reassortment potential of H7N9 influenza virus and other subtypes of influenza A virus

**Figure 3:**
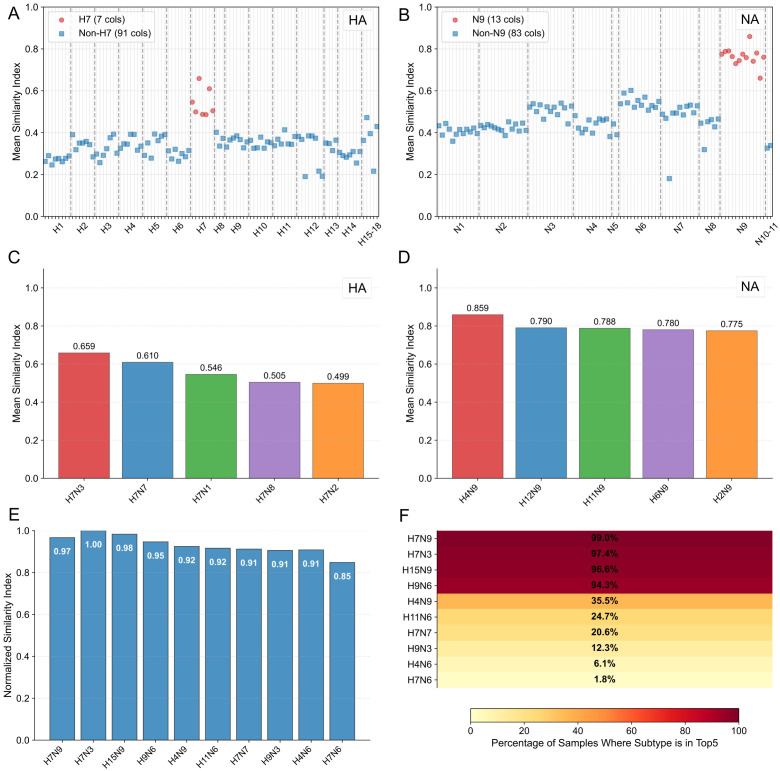
Evaluation of sequence similarity and reassortment potential between H7N9 virus and various subtypes of influenza A virus. **(A)** Similarity Analysis of HA Gene Segment. The average similarity index distribution of the HA gene between H7 subtype (red) and non-H7 subtypes (blue) is shown. The specific high similarity (peak close to 1.0) of H7 subtype in the corresponding segment confirms that the HA gene of H7N9 is highly homologous to the H7 subtype. **(B)** Similarity Analysis of NA Gene Segment. The similarity of the NA gene between N9 subtype (red) and non-N9 subtypes (blue) is compared. The significant high similarity of N9 subtype in the corresponding region confirms the evolutionary conservation among N9 subtypes. **(C)** Distribution of Subtypes with High Similarity in HA Segment. The bar chart shows the top five subtypes with the highest similarity to the HA segment of H7N9. H7N3 ranks first with the highest similarity (0.659), which is consistent with the H7N9-H7N3 reassortment event reported in 2017. **(D)** Distribution of Subtypes with High Similarity in NA Segment. The five subtypes with the highest similarity to the NA segment of H7N9 are shown. N9-related subtypes such as H15N9 stand out, reflecting the subtype-specific conservative characteristics of the NA gene. **(E)** Overall Similarity Ranking of Subtypes with H7N9.The normalized similarity index shows that H7N9 itself has the highest similarity (1.00), followed by H7N3 (0.98) and H15N9 (0.96). The percentage above the bar chart indicates the frequency of the subtype entering the top five in similarity ranking (H7N9: 99.0%, H7N3: 97.4%). **(F)** Heatmap Analysis of Subtypes with High Similarity. The heatmap visualizes the distribution frequency of subtypes in the similarity ranking. The dark-shaded regions are heavily concentrated within H7- and N9-related subtypes, thereby reinforcing the algorithm’s robustness in evaluating reassortment potential.

To evaluate the sequence similarity and reassortment potential of the H7N9 virus relative to other influenza A virus subtypes, this study analyzed nearly 4,000 H7N9 sequences from the GISAID and NCBI databases, along with the first 100 available sequences for each subtype (H1-H18) from NCBI Virus. Following deduplication and quality control, open reading frames (ORFs) were extracted from 1,922 H7N9 viruses using Prodigal. A comprehensive similarity index for all eight gene segments was assessed by calculating Euclidean distance, dot product, and cosine similarity, with weights determined via principal component analysis. Results demonstrated high sequence similarity between the hemagglutinin (HA) segments of H7 subtypes and H7N9, as well as between the neuraminidase (NA) segments of N9 subtypes and H7N9 (**Figures 3A, B**). Subtypes ranking among the top five for overall similarity, such as H7N3 and H4N9 (**Figures 3C, D**), corroborated with the documented 2017 reassortment event where low-pathogenicity H7N9 reassorted with high-pathogenicity H7N3 in avian species, leading to an alkaline amino acid insertion in the HA segment. This concordance strongly validates the algorithm’s efficacy in pinpointing strains with high reassortment potential, further implying that pronounced sequence similarity within phylogenetically related subtypes significantly catalyzes viral reassortment. Furthermore, standardized similarity indices were calculated to quantify sequence similarity between H7N9 and 100 strains of each subtype. H7N3 exhibited the highest index (1.00) and others like H15N9 (0.98) ranged from 0.91 to 0.98, suggesting H7N9’s pronounced similarity to H7- and N9-associated subtypes, likely reflecting genetic conservation (**Figure 3D**). The distribution of sequence similarity was further quantified by computing the average similarity index across all eight segments for each of the 1,922 H7N9 viruses against 100 strains per subtype. A score of 1 was assigned to the top five similar subtypes for each virus, and the percentage of times each subtype ranked in the top five was calculated. H7N9 itself had the highest percentage (99.0%), followed by H7N3 (97.4%) and H15N9 (96.6%) (**Figure 3E**). These results reinforce that H7N9 shares high sequence similarity with cognate or HA/NA-related subtypes, consistent with underlying genetic conservation.

### 3.4 Algorithm validation and genetic analysis of a reassortant influenza strain

**Figure 4:**
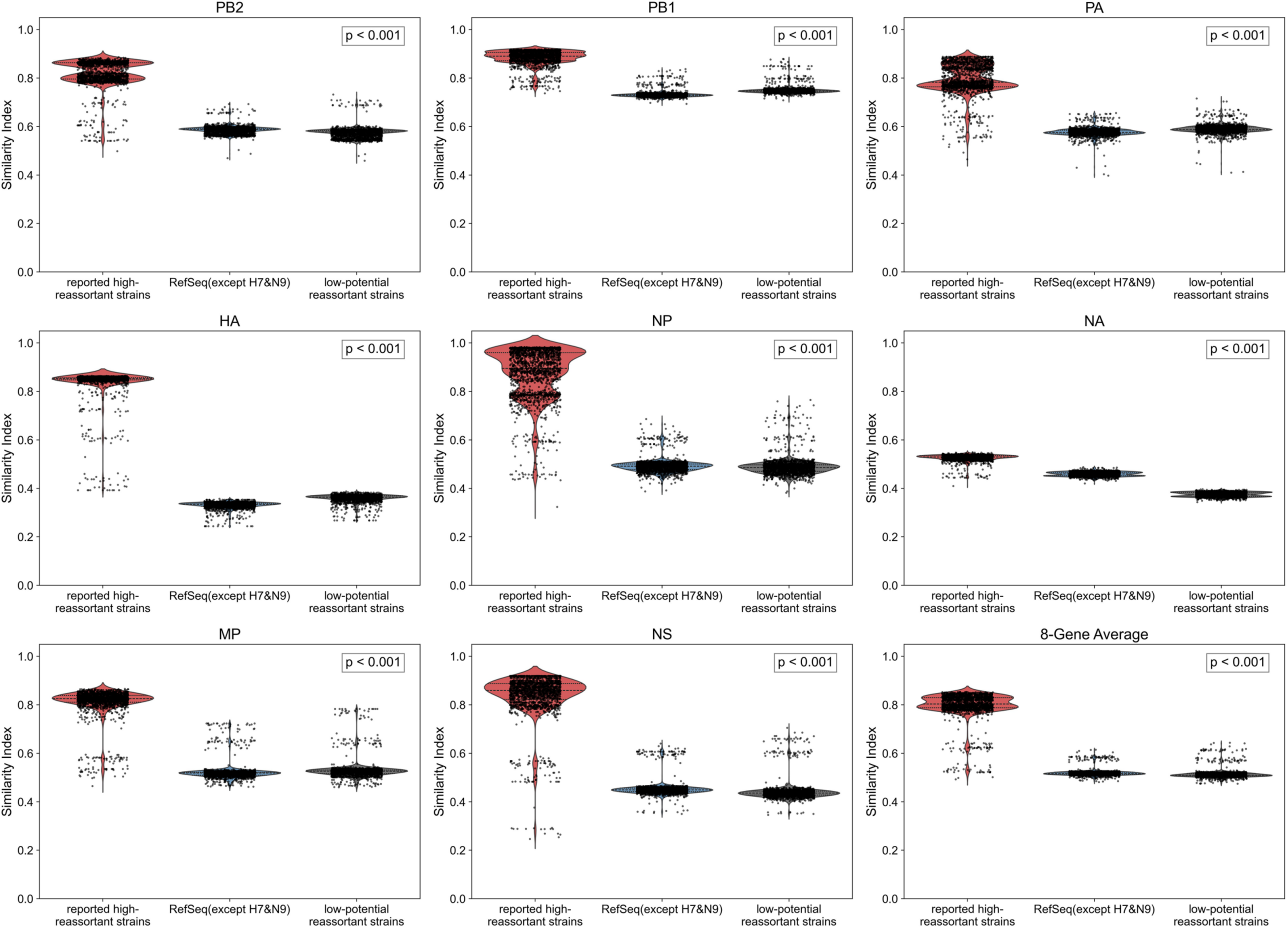
Validation of the algorithm for identifying influenza virus strains with high reassortment potential. Validation of the algorithm was performed by comparing the similarity index distributions of reported high-reassortment potential strains (including A/Shanghai/02/2013 (H7N9), A/chicken/Jilin/SD020/2014 (H7N2), A/chicken/Zhejiang/233/2016 (H7N6), A/chicken/Guangdong/30/2017 (H7N9), and A/Yixing/805/2022 (H3N2)) with reference virus strains (RefSeq, excluding H7 and N9 subtypes) and a low-reassortment potential control group (waterfowl influenza virus strains from North America: A/duck/Alberta/35/1976(H1N1), A/green-winged teal/Wisconsin/7/1974(H3N2), A/mallard/Wisconsin/568/1982(H5N1), A/mallard/California/1418/2013(H5N6), and A/mallard/Wisconsin/426/1979(H8N4)) across eight gene segments (PB2, PB1, PA, HA, NP, NA, MP, NS) and their average. The results demonstrate that the high-reassortment strain group (red) exhibited significantly higher similarity indices and a more concentrated distribution across all tested gene segments compared to the reference group (blue). Furthermore, for all internal gene segments (PB2, PB1, PA, NP, MP, NS), the high-reassortment group also showed significantly higher similarity indices than the low-reassortment group (green) (p< 0.001, Mann-Whitney U test), forming a clear stepwise stratification among the three groups. These collective findings indicate that the algorithm effectively distinguishes influenza virus strains with a high reassortment potential by capturing the underlying genetic architectures and sequence compositional compatibility, rather than merely reflecting natural evolutionary divergence.

To verify the accuracy of the proposed algorithm in identifying H7N9-related strains with high reassortment potential, we selected five reported high reassortment potential strains (including A/Shanghai/02/2013 (H7N9), A/chicken/Jilin/SD020/2014 (H7N2), A/chicken/Zhejiang/233/2016 (H7N6), A/chicken/Guangdong/30/2017 (H7N9), and A/Yixing/805/2022 (H3N2)), calculated their similarity indices with 1922 H7N9 viruses on PB2, PB1, PA, HA, NP, NA, MP, NS gene segments and the average of the 8 genes (using dot product, cosine similarity, and Euclidean distance), and compared them with the similarity indices of reference subtypes of influenza A virus (excluding H7 and N9). The similarity index distribution of high reassortment potential strains was more concentrated in the high-value region on all gene segments, for example, in the PB2 gene, it was concentrated in the 0.6-1.0 range, while the reference group was distributed between 0.4 and 0.8; in the NP gene, the high reassortment group was in 0.4-1.0, and the reference group was in 0.0-0.4; other gene segments such as PB1, PA, HA, MP, NS, and the average of the 8 genes all showed similar trends, with significantly higher similarity indices and more concentrated distribution in the high reassortment group. Statistical tests confirmed that all comparisons had extremely significant differences (p< 0.001, Mann-Whitney U test). To further validate the discriminative ability of the similarity index in reassortment potential assessment, we introduced an additional low-potential control group comprising five early wild waterfowl influenza virus strains from North America ((A/duck/Alberta/35/1976(H1N1), A/green-winged teal/Wisconsin/7/1974(H3N2), A/mallard/Wisconsin/568/1982(H5N1), A/mallard/California/1418/2013(H5N6), and A/mallard/Wisconsin/426/1979(H8N4)) (Austin et al., 1990; Webster et al., 1992; Kawaoka et al., 1998; Krauss et al., 2004), which have no significant gene flow with H7N9 in their evolutionary history and lack ecological, host, or spatiotemporal interfaces with H7N9. Comparative analysis revealed that for all internal gene segments, the high-potential group had significantly higher similarity indices than this low-potential group (p< 0.001). Crucially, the consistently high similarity scores observed in the high-potential group are explicitly driven by their internal gene segments (PB2, PB1, PA, NP, M, and NS). The stark, stepwise stratification among the groups—where the high-potential group scores significantly higher (0.7–0.9) than both the RefSeq background and the North American low-potential controls (0.5–0.6)—effectively eliminates the potential confounding effect of generic phylogenetic distance or “circular reasoning”. Because all strains were evaluated within the exact same mathematical space normalized by the RefSeq baseline, this difference demonstrates that the similarity index does not merely reflect natural evolutionary divergence. Instead, it proves that the algorithm is highly specific and sensitive in capturing the underlying genetic architectures and sequence compositional compatibility essential for high reassortment potential (**Figures 4A–I**). This indicates that our algorithm can effectively distinguish high reassortment potential strains and supports its practicality in reassortment potential assessment of influenza viruses.

**Figure 5:**
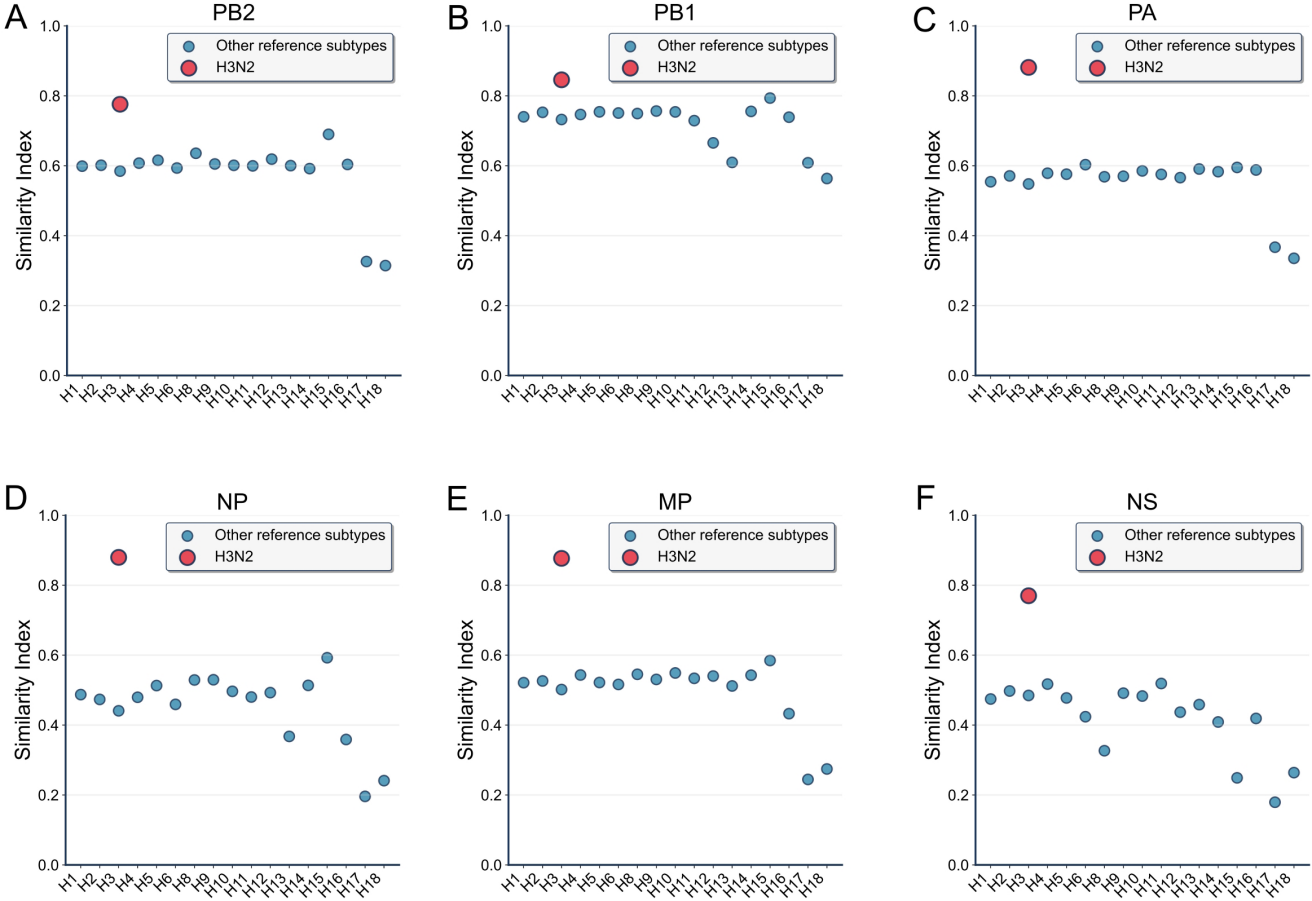
Genetic analysis of the cross-host reassortant strain A/Yixing/805/2022 (H3N2). Similarity indices of the six internal gene segments **(A)** PB2, **(B)** PB1, **(C)** PA, **(D)** NP, **(E)** MP, **(F)** NS between A/Yixing/805/2022 (H3N2) and H7N9 viruses were calculated and compared with reference influenza A virus subtypes (excluding H7 and N9). The A/Yixing/805/2022 strain (red) exhibited consistently high similarity indices (close to 1.0) across all internal gene segments, while reference subtypes (blue) showed significantly lower values (ranging between 0.0–0.8 depending on the segment). These results provide molecular evidence that the internal genes of A/Yixing/805/2022 (H3N2) originate from H7N9 (Clade 2b), supporting its identification as a cross-host reassortant virus associated with fatal encephalitis.

To analyze the genetic characteristics of the A/Yixing/805/2022 (H3N2) strain, which is a cross-host reassortment virus discovered in Yixing, China in August 2022 and whose internal six gene segments (PB2, PB1, PA, NP, MP, NS) originated from H7N9 (Clade 2b) and caused fatal encephalitis, we calculated the similarity indices of the six internal gene segments of this strain with H7N9 viruses and compared them with reference subtypes. The results showed that the similarity indices of the H3N2 strain were higher than those of the reference subtypes on all gene segments: on the PB2 segment, the H3N2 index was concentrated in the high-value region (close to 1.0), while the reference subtypes were distributed lower (around 0.4-0.8); on the PB1 segment, the H3N2 index was high (close to 1.0), and the reference subtypes were lower (around 0.4-0.8); on the PA segment, the H3N2 index was high (close to 1.0), and the reference subtypes were lower (around 0.4-0.8); on the NP segment, the H3N2 index was high (close to 1.0), and the reference subtypes were lower (around 0.0-0.4); on the MP segment, the H3N2 index was high (close to 1.0), and the reference subtypes were lower (around 0.4-0.8); on the NS segment, the H3N2 index was high (close to 1.0), and the reference subtypes were lower (around 0.4-0.8) (**Figure 5**). This suggests that the internal genes of the H3N2 strain are highly similar to H7N9, providing molecular evidence for its cross-host reassortment characteristics.

### 3.5 High-similarity outlier analysis reveals spatiotemporal and evolutionary links between reference influenza A subtypes and H7N9 viruses

**Figure 6:**
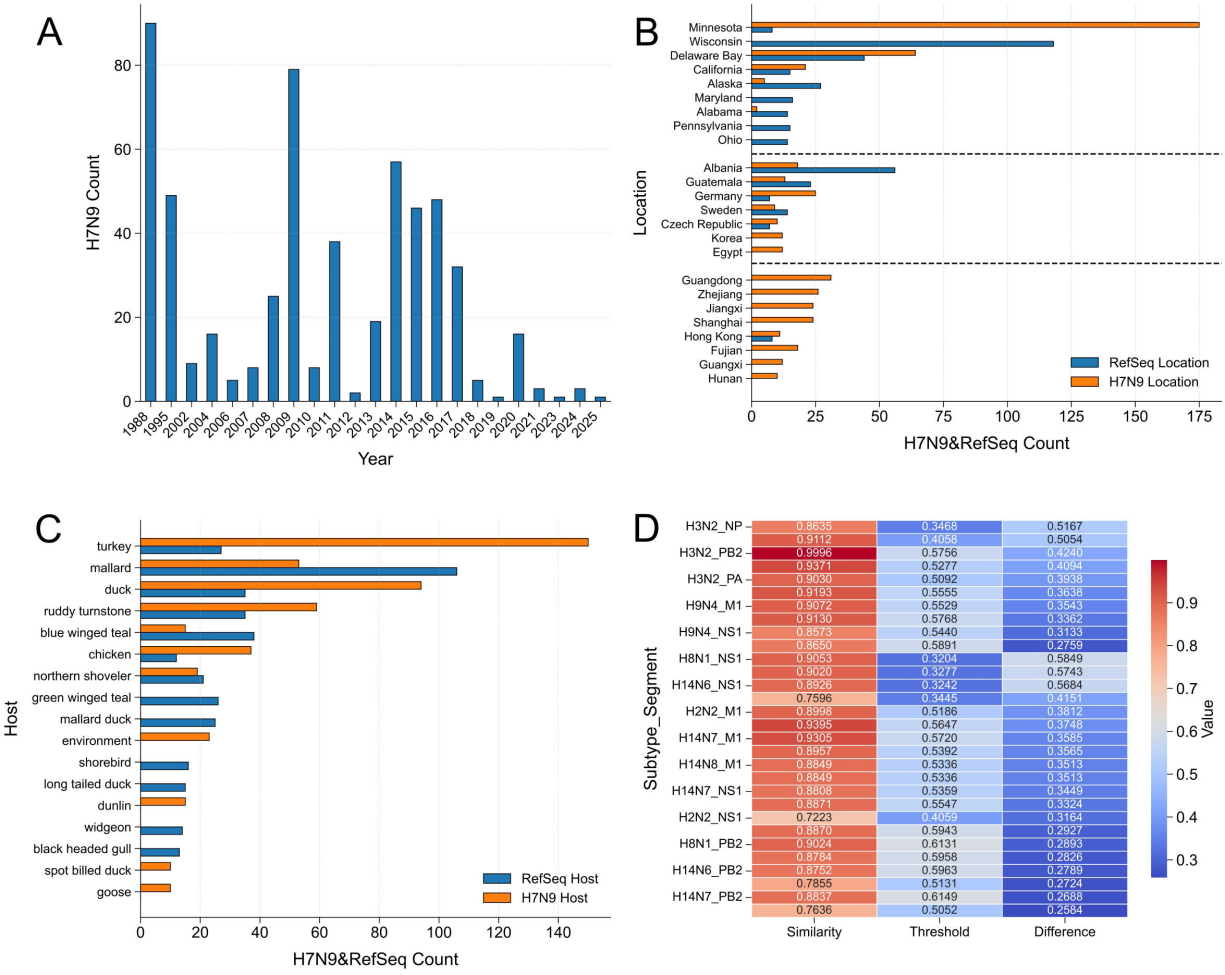
Multidimensional assessment of reassortment potential in H7N9 viruses based on high-similarity outliers. **(A)** Temporal distribution of high-similarity outliers between non-H7N9 influenza A reference sequences and H7N9 viruses.​ A total of 581 outlier data points were identified based on gene sequence similarity analysis. The results demonstrate a significant concentration of high-similarity outlier events during the H7N9 epidemic period (2014–2017), indicating a strong temporal association with the outbreak timeline. **(B)** Spatial co-occurrence of H7N9 viruses and reference influenza A subtypes.​ The bar chart shows concurrent detection of H7N9 (orange) and reference subtypes (blue) across multiple U.S. states, European countries, and Hong Kong, China. This transcontinental spatial coexistence highlights potential geographical hotspots for viral transmission. **(C)** Overlap in host distribution between H7N9 viruses and reference influenza A subtypes. The bar chart shows multiple avian species (e.g., turkey, mallard, domestic duck) hosting both virus types, revealing broad host co-occurrence that may facilitate viral reassortment and transmission. **(D)** Reassortment potential of influenza A reference subtypes with H7N9 viruses based on similarity index differences.​ The heatmap displays the top 30 gene segments with a difference value ≥ 0.25. Subtypes including H3N2, H9N4, H14N6, H14N7, and H8N1 show pronounced similarity differences across multiple segments, indicating elevated reassortment potential with H7N9.

To investigate the genetic high-similarity outliers between non-H7 non-N9 influenza A reference sequences and H7N9 viruses, we analyzed the outlier points of reference sequences shown in **Figure 4**. Similarity indices were computed for each gene segment, and a threshold for identifying high-similarity outliers was strictly defined using the upper bound of the interquartile range method (calculated as Q3 + 1.5 × IQR, where Q3 is the 75th percentile and IQR is the interquartile range of the similarity index distribution). This led to the identification of 581 outlier points exceeding this threshold. The temporal distribution of these outliers exhibited a sustained escalation from 2014 to 2017, seamlessly aligning with the H7N9 outbreak waves and indicating strong temporal synchronization (**Figure 6A**). Spatially, simultaneous detections of H7N9 and reference subtypes were observed across multiple transcontinental regions, including several U.S. states, European countries, and Hong Kong, China, highlighting potential geographic hotspots for viral transmission and reassortment (**Figure 6B**). In addition, host distribution analysis revealed broad overlaps across avian species such as turkeys, mallards, and chickens, suggesting that these hosts represent key ecological interfaces for co-infection (**Figure 6C**). To evaluate reassortment potential, we calculated the difference in similarity indices exceeding the threshold and retained entries with a difference ≥0.25 (124 entries in total). Subsequent analysis identified subtypes such as H3N2—which exhibited high frequency and substantial differences across multiple segments, corroborating its documented reassortment with H7N9—as well as H9N4, H14N6, H14N7, and H8N1 (**Figure 6D**). These subtypes displayed marked similarity differences, indicating their potential involvement in future reassortment events and providing critical insights for influenza risk assessment.

## 4 Discussion

Cross-species transmission and reassortment evolution of IAV are key drivers of global influenza pandemics. Most IAV subtypes originate from avian influenza viruses, posing a grave threat to public health (Shu and Mccauley, 2017). When different IAV subtypes co-circulate in the same region, the high genetic diversity of their eight-segment RNA genomes leads to high-frequency genetic recombination and high mutation rates, thereby yielding reassortant strains with novel HA/NA combinations, which significantly amplifies the risk of cross-species infection (Zhang et al., 2019). Conventional epidemiological surveillance predominantly relies on retrospective case reports, laboratory diagnostics, and expert consensus, which inherently limits the capacity to accurately forecast viral evolutionary trajectories and latent reassortment potential. Obvious limitations in prevention and control were indicated, particularly for highly variable viruses such as IAV (Zhou et al., 2022).

In recent years, the application of artificial intelligence technology in the field of infectious disease modeling has provided a new paradigm for viral risk prediction (Kraemer et al., 2025). Based on the biological characteristics of IAV and the principles of mathematical vector calculation, our team proposed a Similarity Index evaluation method that does not require sequence alignment: viral gene sequences are encoded into high-dimensional vectors through algorithms, combined with multi-dimensional quantitative analysis of Dot Product, Cosine Similarity, and Euclidean Distance, and the Similarity Index is constructed after Principal Component Analysis (PCA) optimization to achieve quantitative evaluation of the correlation and reassortment potential between strains.

To verify the reliability of the method, this study selected five previously validated high-potential strains (A/Shanghai/02/2013 (H7N9) (Gao et al., 2013), A/chicken/Guangdong/30/2017 (H7N9) (Ke et al., 2017), A/Yixing/805/2022 (H3N2) (Wang et al., 2025), A/chicken/Jilin/SD020/2014 (H7N2) (Qiu et al., 2019; Shi et al., 2014), A/chicken/Zhejiang/233/2016 (H7N6) (Zhao et al., 2019)) for parallel verification, ensuring that the algorithm meets scientific requirements both in mathematical logic and biological practice. Furthermore, combined with the temporal, spatial, and host distribution characteristics of H7N9 high-risk spillover strains and the Similarity Index, the evolutionary law of H7N9 reassortment potential was systematically analyzed, providing data support and method innovation for influenza prevention and control, as well as new research ideas for the field of infectious disease risk prediction.

Comprehensive analysis of **Figure 1** and **Figure 6** enables systematic interpretation of the temporal, spatial, and host distribution characteristics of H7N9 and its high-similarity spillover strains, providing multi-dimensional basis for risk assessment and monitoring. Specifically, the core distribution characteristics of H7N9 presented in **Figure 1** show that since it was identified as a novel IAV in 2013, the virus has rapidly caused large-scale epidemics (Gao et al., 2013), with five outbreaks occurring between 2013 and 2017, during which high-risk strains continued to emerge (Wang et al., 2017); in early 2017, H7N9 further reassorted into a highly pathogenic strain (HP-H7N9), with multiple basic amino acids inserted at its HA cleavage site, increasing the mortality rate to over 50% and resulting in limited human-to-human transmission (Ke et al., 2017). The sporadic emergence of high-risk strains in recent years further highlights the persistence of its epidemic risk. Regarding spatial dynamics, there is pronounced geographical heterogeneity in the detection of H7N9 in China. Southern and eastern provinces (Guangdong, Anhui, Zhejiang, Jiangsu, etc.) act as primary epidemiological epicenters, where the number of detections is significantly higher than those in northern regions (Li et al., 2015); globally, the distribution of the virus is extremely uneven, with China accounting for 96.2% (3028/3148) of all reported strains, and only sporadic cases in countries such as the United States and Japan. This pattern clearly indicates that China is the main epidemic area of H7N9 (Huo et al., 2017). At the host level, H7N9 is not only frequently detected in human (Su et al., 2017) and environmental samples (Yang et al., 2018) but also shows significant epidemic characteristics in avian hosts such as mallards, domestic chickens, and turkeys, reflecting its potential for cross-species transmission (Wang et al., 2014).

On this basis, the further analysis of H7N9 high-similarity spillover strains in **Figure 6** further expands the risk dimension of the above distribution characteristics: spatially, H7N9 and spillover strains have been co-detected in multiple regions, including Minnesota, Delaware Bay, California in the United States, countries such as Albania, Germany, and Sweden, and the Hong Kong region of China. This spatial overlaps in specific time and space essentially provides geographical interface conditions for viral reassortment—among them, H7N9 is detected most frequently in Minnesota, while spillover strains are concentrated in Wisconsin. In China, the co-detection hotspots are still concentrated in southern provinces such as Guangdong and Zhejiang, which echoes the overall epidemic characteristics of H7N9. Host overlap analysis shows that species such as turkeys, mallards, domestic ducks, ruddy turnstones, blue-winged teal, and chickens carry both H7N9 and spillover strains, and such co-infected hosts are highly likely to become key ecological niches for viral reassortment and transmission (Sanjuán and Domingo-Calap, 2016); analysis of host preference further indicates that the H7N9 virus is mainly found in turkeys and wild ducks, which provides a targeted basis for precise monitoring at the host level.

Combined with the above temporal, spatial, and host distribution characteristics, subsequent monitoring focused on two types of key risk scenarios: spatially, it is necessary to focus on cross-regional distribution overlap zones (such as some regions in China and the United States, multiple European countries, and southern provinces of China) to guard against the cross-border or cross-regional importation and spread of the virus through these geographical interfaces; at the host level, it is necessary to strengthen the regular monitoring of co-infected species such as turkeys, wild ducks, and domestic chickens to block the ecological chain of viral reassortment. This reassortment potential of “wild bird-derived HA/NA subtypes + H9N2 internal genes prevalent in poultry” is not a theoretical deduction, as evidenced by the epidemic characteristics of H5N6 virus (Shen et al., 2016): The host range of H5N6, which includes poultry such as chickens and ducks, mirrors that of H7N9. Moreover, both subtypes have acquired their internal gene cassettes from H9N2 viruses widely circulating in poultry. This underscores the role of H9N2 as an “internal gene engine,” capable of conferring mammalian adaptability to diverse HA/NA subtype configurations. From a biological perspective, the exceptionally high Similarity Index (SI) scores driven by these internal genes are not merely a reflection of evolutionary homology, but a quantitative manifestation of underlying structural and functional compatibility. Our alignment-free algorithm, which vectorizes global k-mer frequencies and codon pair usage biases, effectively captures the sequence-level prerequisites for the efficient assembly of the viral ribonucleoprotein (vRNP) complex (composed of PB2, PB1, PA, and NP). High compatibility in codon usage and nucleotide composition ensures optimal transcription, replication, and translation efficiencies when these segments are reassorted into a new genomic background. Therefore, the algorithm’s high scoring of these internal segments objectively mirrors the biological reality: it detects the highly compatible genetic foundation that allows the H9N2 “engine” to successfully support the survival and efficient replication of novel, potentially pandemic reassortants. H5N6 not only possesses the high pathogenicity of H5 subtype to poultry but also acquires mammalian adaptability conferred by H9N2, and its risk characteristics are highly convergent with those of H7N9. It is therefore plausible to deduce that analogous reassortment paradigms will persistently drive the genesis of novel high-risk strains. In the future, it is necessary to continuously monitor the reassortment events between novel HA/NA subtypes carried by wild birds and H9N2 virus in poultry to early warn of potential pandemic risks.

This study first completed the verification of algorithm reliability through multi-dimensional analysis and then carried out potential risk prediction based on the verified algorithm, with the core logic of “from reliability support to precise prediction.”

At the algorithm verification level: HA/NA gene typing analysis in **Figure 3** shows that the Similarity Index of H7 lineage strains is significantly higher than that of non-H7 lineages, and the Similarity Index of N9 lineage strains is significantly higher than that of non-N9 lineages, which are consistent with the genetic evolution laws of IAV HA and NA genes, respectively, initially confirming the rationality of the algorithm; further comprehensive Similarity Index analysis in **Figures 3E, F** indicates that H7N9 strains account for the highest proportion among all reference subtypes and most belong to H7 or N9 lineages, which is highly consistent with the biological classification characteristics of H7N9, verifying the accuracy of the algorithm. Notably, the H9N6 strain of non-H7/non-N9 lineage has a top 5 proportion of comprehensive Similarity Index with H7N9 reaching 94.3% and a normalized Similarity Index of 0.95, which also provides an early warning signal for potential high-risk reassortment events. On this basis, **Figure 4** selected five previously reported high-potential strains (including 2 typical H7N9 strains and 3 H7N9-related high-risk strains) for inter-group difference analysis with reference subtypes excluding H7 and N9 lineages. The P-values of all comparison groups are< 0.001, indicating that the algorithm results are completely consistent with the biologically verified facts of previous studies; among them, the non-H7/non-N9 strain A/Yixing/805/2022 (H3N2) that caused human encephalitis cases in 2022 was further confirmed for its high similarity and reassortment association with H7N9 through separate verification in **Figure 5**. Multiple verification results fully guarantee the reliability of the algorithm.

Based on the above verified reliable algorithm, **Figure 6** carried out the prediction of high-potential strains and potential reassortant subtypes: similarity ranking results show that among the 100 reference subtypes excluding H7 and N9 lineages, H3N2 has the highest similarity with H7N9. This conclusion is mutually confirmed with the outbreak fact of the Yixing H3N2 reassortant strain in 2022, reflecting the practical value of the prediction model; at the same time, subtypes such as H9N4, H14N6, H14N7, and H8N1 exhibit multi-segment high similarity characteristics with H7N9, suggesting that these subtypes may become key participants in future reassortment events. At the segment level, the M1 and NS1 segments of H9N4, the NS1 and PB2 segments of H8N1, and the NS1 and PB2 segments of H14N6 have particularly prominent similarity with H7N9. The proteins encoded by these segments are involved in key processes such as viral replication, transcription, and immune escape, and their reassortment may significantly change the pathogenicity and transmission ability of the virus (Wang et al., 2014). This result also provides an important basis for subsequent targeted monitoring at the gene level.

Although this study achieved quantitative evaluation of H7N9 reassortment potential, there are still the following limitations. Initially, limitation of sample size of reference subtypes: only one strain was selected as a representative for each reference subtype. Although this ensures the unity of reference standards, it may introduce deviations due to the genetic specificity of a single strain. Moreover, the number of complete 8-segment sequences of most reference subtypes in the NCBI Virus database is extremely limited (single-digit or even only one strain), making it difficult to implement multi-strain repeated verification. Additionally, while selecting reference strains based on the ‘earliest collection time’ establishes a necessary ancestral baseline for source-tracing, it inherently introduces a temporal gap. Given the high mutation rates of influenza viruses, this temporal difference might naturally influence the calculated genetic distance. Future studies incorporating temporally diverse reference strains are warranted to further validate and refine the Similarity Index. Finally, regarding the impact of sequence completeness: a subset of the 100 reference subtypes yielded only 7 valid open reading frames (ORFs), resulting in the lack of a unified 8-segment basis for the calculation of comprehensive Similarity Index, which may affect the rigor of the results.

The three core advantages of the Similarity Index evaluation method proposed in this study can be summarized as follows. (1) Universal applicability: it is applicable to H7N9 and all IAV strains. As long as complete gene sequences are obtained, the Similarity Index can be calculated to achieve rapid evaluation of correlation and reassortment potential; (2) Data quantifiability: it converts abstract viral genetic associations into specific values, supporting horizontal comparison between different strains and subtypes, and improving the objectivity and accuracy of risk assessment; (3) No need for sequence alignment: it avoids the alignment problem caused by the high variability of IAV (especially H7N9) sequences (Lam et al., 2013), with simple operation and strong reproducibility, significantly improving analysis efficiency.

Future research will proceed in three principal directions. First, it will expand the dataset beyond the currently included 98,787 IAV strains to improve subtype representation and uncover additional high-risk reassortment combinations. Second, the algorithmic framework will be optimized by integrating viral biological characteristics (such as protein structure and functional domain distribution) with mathematical principles to refine distance metrics and weighting approaches, thereby enhancing the accuracy of risk prediction. Lastly, application scenarios will be broadened through the development of a multidimensional risk prediction system that incorporates host tropism, cross-species infection mechanisms, and virulence-related genetic markers, ultimately supporting a comprehensive analysis of IAV evolution and strengthening global preparedness and control efforts.

## 5 Conclusion

In summary, our alignment-free computational framework provides a holistic and quantitative evaluation of the H7N9 reassortment landscape. This work not only confirms the substantial reassortment potential of pivotal historical strains but also identifies future candidate viruses with high reassortment likelihood. These findings highlight the dynamic and persistent threat of influenza virus evolution. The proposed methodology and insights are therefore essential for shaping targeted surveillance and proactive public health interventions against emerging pandemic strains.

## Data Availability

The original contributions presented in the study are included in the article/**Supplementary Material**. Further inquiries can be directed to the corresponding authors.
